# The Meeting of the American Medical Association at Richmond

**Published:** 1852-05

**Authors:** 


*The Meeting of the American Medical Association at Richmond.*

THIS Association has recently held its 6th annual meeting. The numbers in attendance were somewhat less than on most former occasions. As the practical working of the constitutional change effected at Charleston was, for the first time, to be tested, this meeting was one of unusual interest. That change consisted in the abolition of the standing committees, and substituting in their stead nearly thirty special committees. Of these, not more than a fourth part made reports; some requesting to be discharged, and others, we believe, took no notice of their appointment. It can, therefore, scarcely be said that the new plan has had more than very partial success.

The objects of the Association divide themselves into two branches, 1st, Scientific; 2d, Medical Politics.

In reference to the first, it is difficult to say what will be the result of this meeting. The committee on Prize Essays reported that, of fifteen submitted to their examination, but one was deemed worthy of a prize. This, the subject of which was, The Different Tones of the Sounds perceived in Auscultation, and their value as diagnostic signs,—was from the pen of Prof. Flint, of Buffalo, whose name is a sufficient guarantee of its merit.

Of the different reports it would be unjust to speak from simply listening, as circumstances did not admit of their being clearly heard.

Of the papers offered for reading still less is known, as they were mostly referred to the committee on publication without being heard.

We must await, therefore, the volume of transactions to decide upon the scientific part of the action of this meeting.

In regard to the questions of "medical reform," our information is much more clear. The time of the Association was, in a great measure, taken up in the discussion of proposed amendments of the constitution. These turned mainly on the views published by Dr. Samuel Jackson, of Philadelphia, which are, in short, the turning out of professors, hospital physicians and surgeons as delegates, and admitting all members of county societies as members of the Association.

It seems to be clear to the mind of this philosopher that a life spent in teaching, or hospital practice, disqualifies for the high duty of deliberating on the best interests of the profession, and of sustaining the national character of the Association by contributions to its volumes of published "transactions."

The special committee to whom this subject was referred, adopted these views only in part, proposing to cut off all lunatic asylums and hospitals with less than 100 beds from representations, also all schools with less than six professors, "*except the University of Virginia*," which has four, and diminish the number of delegates to which colleges and other hospitals are entitled, to one instead of two, as at present. This lies over till next year. Of the two plans, that of Dr. Jackson is by far the preferable, as being frank, plain and straight-forward, and effecting at once what the other proposes to do indirectly and by halves. It is also free from such invidious distinctions as the latter proposes. The exception of the University of Virginia is nominally founded on the peculiarity of their mode of teaching, which, being by lectures and examinations, does not differ sensibly from the plan pursued throughout the Union. Why they should be excepted from a rule that will apply to any other institution which shall adopt the same plan, and lecture the same length of time, we leave to its friends to explain.

To every one acquainted with the organizations of county societies and State societies, and their general decline, it is obvious that the adoption of the plan proposed involves the existence of the Association.

As we propose to return to this subject often during the year, we shall not dwell further upon it here.

New York was selected as the next place of meeting. In making this selection the Association reversed the decision of their own nominating committee, which recommended St. Louis. The West is justly entitled to the meeting every third year; but the small number of delegates it sends prevents it from making its just claims heard in this respect. Practically speaking, it has little interest in the present Association, and we suggest to our brethren of the press and profession, whether the formation of a western association, whose objects should be exclusively scientific, and which should in no wise interfere with the eastern society, would not be desirable.

It is humiliating to witness, that while the National Association for the advancement of Science is invited to meet at different cities, —its members carried and entertained, and their proceedings published, without charge, the Association for the advancement of Medical Science is looked upon only as a combination for the maintenance of the interests of a class, and the promotion of the private objects of its members.

Can it not by its acts free itself from so low a position in the public mind?

It would be unpardonable to close any notice of the meeting at Richmond without an acknowledgment of the generous and noble hospitality of the citizens and the profession of the State and city. Every institution was thrown open to delegates, and every attention shown calculated to make their visit delightful. D. B.

